# Accurate prediction of protein-lncRNA interactions by diffusion and HeteSim features across heterogeneous network

**DOI:** 10.1186/s12859-018-2390-0

**Published:** 2018-10-11

**Authors:** Lei Deng, Junqiang Wang, Yun Xiao, Zixiang Wang, Hui Liu

**Affiliations:** 1 School of Software, Central South University, Changsha, 410075 China; 2 Lab of Information Management, Changzhou University, Jiangsu, 213164 China

**Keywords:** Protein-lncRNA interaction, Heterogenous network, HeteSim score, Gradient tree boosting

## Abstract

**Background:**

Identifying the interactions between proteins and long non-coding RNAs (lncRNAs) is of great importance to decipher the functional mechanisms of lncRNAs. However, current experimental techniques for detection of lncRNA-protein interactions are limited and inefficient. Many methods have been proposed to predict protein-lncRNA interactions, but few studies make use of the topological information of heterogenous biological networks associated with the lncRNAs.

**Results:**

In this work, we propose a novel approach, PLIPCOM, using two groups of network features to detect protein-lncRNA interactions. In particular, diffusion features and HeteSim features are extracted from protein-lncRNA heterogenous network, and then combined to build the prediction model using the Gradient Tree Boosting (GTB) algorithm. Our study highlights that the topological features of the heterogeneous network are crucial for predicting protein-lncRNA interactions. The cross-validation experiments on the benchmark dataset show that PLIPCOM method substantially outperformed previous state-of-the-art approaches in predicting protein-lncRNA interactions. We also prove the robustness of the proposed method on three unbalanced data sets. Moreover, our case studies demonstrate that our method is effective and reliable in predicting the interactions between lncRNAs and proteins.

**Availability:**

The source code and supporting files are publicly available at: http://denglab.org/PLIPCOM/.

## Background

Long non-coding RNAs (lncRNAs) have been intensively investigated in recent years [1, 2], and show close connection to transcriptional regulation, RNA splicing, cell cycle and disease. At present, a great majority of lncRNAs have been identified, but their functional annotations verified by experiment remains very limited [3, 4]. Recent studies have proved that the function of lncRNAs strikes a chord with the corresponding binding-proteins [5–7]. Therefore, the binding proteins of lncRNAs are urgent to be uncovered for better understand of the biological functions of lncRNAs.

Although high-throughput methods for characterization of protein-RNA interactions have been developed [8, 9], *in silico* methods are appealing for characterization of the lncRNAs that are less experimentally covered due to technical challenge [10]. One common way for computationally predicting lncRNA-binding proteins is based on protein sequence and structural information. For example, Muppirala et al. [11] developed a computational approach to predict lncRNA-protein interactions by using the 3-mer and 4-mer conjoint triad features from amino acid and nucleotide sequences to train a prediction models. Wang et al. [12] used the same data set by Muppirala et al. [11] to develop another predictor based on Naive Bayes (NB) and Extended Naive Bayes (ENB). Recently, Lu et al. [13] presented lncPro, a prediction method for Protein-lncRNA associations using Fisher linear discriminant approach. The features used in lncPro consist of RNA/protein secondary structures, hydrogen-bonding propensities and Van der Waals’ propensities.

In recent years, network-based methods have widely been used to predict lncRNA functions [14, 15]. Many studies have paid attention to integration of heterogeneous data into a single network via data fusion or network-based inference [16–21]. The network propagation algorithms, such as the Katz measure [22], random walk with restart (RWR) [23], LPIHN [24] and PRINCE [25, 26], have been used to investigate the topological features of biomolecular networks in a variety of issues, such as disease-associated gene prioritization, drug repositioning and drug-target interaction prediction. Random Walk with Restart (RWR) [23] is widely used for prioritization of candidate nodes in a weighted network. LPIHN [24] extends the random walk with restart to the heterogeneous network. PRINCE [25, 26] formulates the constraints on prioritization function that relate to its smoothness over the network and usage of prior information. Recently, we developed PLPIHS [27], which uses the HeteSim measure to predict protein-lncRNA interactions in the heterogeneous network.

In this paper, we introduced an computational approach for protein-lncRNA interaction prediction, referred to as PLIPCOM, based on protein-lncRNA heterogeneous network. The heterogeneous network is constructed from three subnetworks, namely protein-protein interaction network, protein-lncRNA association network and lncRNA co-expression network. PLIPCOM incorporates (i) low dimensional diffusion features calculated using random walks with restart (RWR) and a dimension reduction approach (SVD), and (ii) HeteSim features obtained by computing the numbers of different paths from protein to lncRNA in the heterogeneous network. The final prediction model is based on the Gradient Tree Boosting (GTB) algorithm using the two groups of network features. We compared our method to both traditional classifiers and existing prediction methods on multiple datasets, the performance comparison results have shown that our method obtained state-of-the-art performance in predicting protein-lncRNA interactions.

It is worth noting that we have substantially extended and improved our preliminary work published on the BIBM2017 conference proceeding [28]. The improvements include: 1) We presented more detail of the methodology of PLIPCOM, such as the construction of protein-lncRNA heterogenous work, feature extraction and gradient tree boosting algorithm; 2) We have conducted extensive evaluation experiments to demonstrate the performance of the proposed method on multiple data sets with different positive and negative sample ratios, i.e. P:N=1:1,1:2,1:5,1:10, respectively. Particularly, we compared PLIPCOM with our previous method PLPIHS [27] on four independent test datasets, and the experimental results show that PLIPCOM significantly outperform our previous method; 3) To verify the effectiveness of the diffusion and HeteSim features in predicting proteinlncRNA interactions, we evaluated the predictive performance of the two types of features alone and combination of them, on the benchmark dataset; 4) Case studies have been described to show that our method is effective and reliable in predicting the interactions between lncRNAs and proteins; 5) Last but not the least, we have conducted the time complexity analysis of PLIPCOM.

## Methods

### Overview of PLIPCOM

**Fig. 1:**
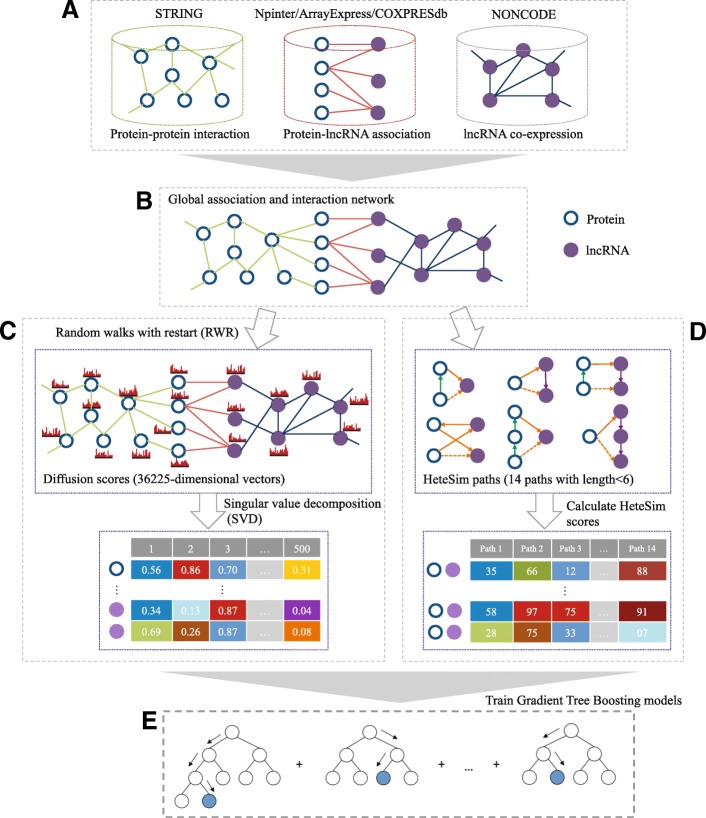
Flowchart of PLIPCOM consists of five steps. **a** Protein-protein interaction, protein-lncRNA association, and lncRNA co-expression data are extracted from multiple public databases. **b** Global heterogeneous network is built by integrating three subnetworks. **c** The diffusion scores are calculated using random walks with restart (RWR) on the heterogeneous network, and then dimensionality reduction is conducted to obtain low-dimensional topological features using singular value decomposition (SVD). **d** For each lncRNA-protein pair, the HeteSim scores are calculate by counting the numbers of different paths linking them on the heterogeneous network. **e** The diffusion features and HeteSim features are combined to train the Gradient tree boosting (GTB) classifier for predicting protein-lncRNA interactions

As shown in Fig. 1, the PLIPCOM framework consists of five steps. (A) Collection of three types of data sources, including protein-protein interaction network, protein-lncRNA associations and lncRNA co-expression network. (B) Construction of the global heterogenous network by merging the three networks. (C) Running random walks with restart (RWR) in the heterogeneous network to obtain a diffusion state for each node, which captures its topological relevance to all other nodes (proteins and lncRNAs) in the network. We further apply the singular value decomposition (SVD) to conduct dimension reduction and obtained a 500-dimensional feature vector for each node in the network. (D) The HeteSim score is a measure to estimate the correlation of a pair of nodes relying on the paths that connects the two nodes through a string of nodes. We computed 14 types of HeteSim features from protein-lncRNA heterogenous network. (E) We integrate the 1000-dimension (500-dimensional for the protein and 500-dimensional for the lncRNA) diffusion features and 14-dimension HeteSim scores to train the protein-lncRNA interaction prediction model using gradient tree boosting (GTB) algorithm.

### Data sources

#### Protein-protein interaction

All human lncRNA genes and protein-coding genes were obtained from GENCODE database [29] (Release 24), which includes 15,941 lncRNA genes and 20,284 protein-coding genes. We obtained the human protein-protein interactions (PPIs) from STRING database [30] (V10.0), which collected PPIs from high-throughput experiments, as well as computational predictions and text mining results. A total of 7,866,428 human PPIs are obtained.

#### LncRNA-lncRNA co-expression

We downloaded the expression profiles of lncRNA genes from NONCONDE 2016 database [31], and calculated the lncRNA co-expression similarity between each two lncRNAs using Pearson’s correlation coefficient.

#### Protein-lncRNA association

We obtained the protein-lncRNA interactions from NPinter v3.0 [32], which contains 491,416 experimentally verified interactions. In addition to the known proteinlncRNA interactions, we also employed the co-expression profiles to build the protein-lncRNA association network. In particular, three co-expression datasets (Hsa.c4-1, Hsa2.c2-0 and Hsa3.c1-0) with pre-computed pairwise Pearson correlation coefficients from COXPRESdb database [33] were downloaded. The three correlations are then integrated as below:

1 $$ C(l,p)=1-\prod_{d=1}^{D}(1-C_{d} (l,p))\ \ if ~C_{d} (l,p)>0  $$

where *C*(*l*,*p*) is the integrative correlation coefficient between lncRNA *l* and protein-coding gene *p*, *C*_*d*_(*l*,*p*) represents the correlation coefficient between *l* and *p* in dataset *d*, and *D* is the number of data sets. In particular, we take into account the gene pairs whose correlation coefficient are positive, and discard those with negative correlation coefficients, as the mutual exclusion relationship indicates that protein is unlikely to interacting with the lncRNA.

An additional paired-end RNA-seq datasest including 19 human normal tissues are obtained from the Human Body Map 2 project (ArrayExpress accession E-MTAB-513) and another study (GEO accession no.GSE30554). Expression levels are calculated using Tophat and cufflinks, and the co-expressions of proteinlncRNA pairs are evaluated using Pearson’s correlation coefficients.

Finally, we built a global heterogenous network by merging the three types of subnetworks (protein-protein interaction network, lncRNA-lncRNA co-expression network, and protein-lncRNA association network). The resulting network has 36,225 nodes (15,941 lncRNAs and 20,284 proteins) and 2,339,152 edges after removal of edges wit similarity scores <0.5.

### Low-dimensional network diffusion features

The diffusion feature is a high-dimensional vector describing the topological properties of each node, which captures its relevance to all other nodes in the network. The network diffusion features can be calculated using random walk with restart (RWR) algorithm [34, 35] on the global heterogenous network. RWR is able to identify relevant or similar nodes by taking the local and global topological structure within the network into account. Let *G* denote the adjacency matrix for the global network, and *T* represent the transition probability matrix. Each entry *T*_*ij*_ holding the transition probability from node *i* to node *j* is computed as below

2 $$ {T_{ij}} = \frac{{{G_{ij}}}}{{{\sum\nolimits}_{k} {{G_{ik}}} }},  $$

in which *G*_*ij*_ is equal to 1 if node *i* is connected to node *j* in the network, and 0 otherwise. The RWR process can be written as follows:

3 $$ {P_{t + 1}} = (1 - \alpha)T{P_{t}} + \alpha {P_{0}},  $$

where *α* is the restart probability leveraging the importance of local and global topological information; *P*_*t*_ is a probability distribution whose *i*-th element represents the probability of node *i* being visited at step *t*. After enough number of iterations, RWR will converge so that *P*_*t*_ holds the stable diffusion distribution. If two nodes have similar diffusion states, they locate in similar situation within the global network with respect to other nodes. Since there are 36,225 nodes (15,941 lncRNA nodes and 20,284 protein nodes) in the network, each node has a 36,225-dimensional diffusion state.

In view of excessively high-dimensional features are prone to noise interference and time-consuming in model training, we apply singular value decomposition (SVD) [36–38] to reduce the dimensionality of the diffusion features derived by RWR. Formally, the probability transition matrix *P* is factorized into the form as below:

4 $$ P = U\Sigma V,  $$

where the diagonal entries of *Σ* are the singular values of *P*, and the columns of *U* and *V* are the left-singular vectors and right-singular vectors of *P*, respectively. For a given number *n* of output dimensions, we assign the top *n* columns of *Σ*^1/2^*V* to *x*_*i*_, namely,

5 $$ X = \Sigma^{1/2}V,  $$

where *X* is the derived low-dimensional feature matrix from the high-dimensional diffusion features. In this work we set *n*=500 according to previous study [38].

### HeteSim score-based features

The HeteSim score is a measure to estimate the correlation of a pair of nodes, and its value depends on the paths that connects the two nodes through a string of nodes in a graph [39]. HeteSim score can be easily extended to calculate the relevance of nodes in a heterogenous network. Denote by *L* and *P* two kinds of nodes in a heterogenous network, (*A*_*LP*_)_*n*∗*m*_ is an adjacent matrix, the normalization matrix of *A*_*LP*_ with respect to the row vector is defined as

6 $$ A_{LP}(i,j)=\frac{A_{LP}(i,j)}{\sum_{k=1}^{m}A_{LP}(i,k)}.  $$

The reachable probability matrix $R_{\mathcal {P}}$ can be defined as:

7 $$ R_{\mathcal{P}}=A_{P_{1}P_{2}}A_{P_{2}P_{3}}\cdots A_{P_{n}P_{n+1}}  $$

where $\mathcal {P}=(P_{1}P_{2}\cdots P_{n+1})$ represents the set of paths of length *n*, and *P*_*i*_ belongs to any nodes in the heterogenous network.

The detailed calculation procedure can be found in our previous work [27]. Here we calculate the paths from a protein to a lncRNA in the heterogenous network with. As listed in Table 1, there are in total 14 different paths from a protein to a lncRNA under the constraint of length <6. So, we obtain a 14-dimensional HeteSim feature for each node in the heterogenous network.

**Table 1:** 14 different paths from a protein to a lncRNA with length less than 6 in the heterogenous network

ID	name	path
1	PLL	protein-lncRNA-lncRNA
2	PPL	protein-protein-lncRNA
3	PPLL	protein-protein-lncRNA-lncRNA
4	PLPL	protein-lncRNA-protein-lncRNA
5	PLLL	protein-lncRNA-lncRNA-lncRNA
6	PPPL	protein-protein-protein-lncRNA
7	PPPPL	protein-protein-protein-protein-lncRNA
8	PLPPL	protein-lncRNA-protein-protein-lncRNA
9	PPLPL	protein-protein-lncRNA-protein-lncRNA
10	PLLPL	protein-lncRNA-lncRNA-protein-lncRNA
11	PPPLL	protein-protein-protein-lncRNA-lncRNA
12	PLPLL	protein-lncRNA-protein-lncRNA-lncRNA
13	PPLLL	protein-protein-lncRNA-lncRNA-lncRNA
14	PLLLL	protein-lncRNA-lncRNA-lncRNA-lncRNA

### The gradient tree boosting classifier

Based on the derived diffusion and HeteSim features, we build a classifier using the gradient tree boosting (GTB) [40] algorithm to predict protein-lncRNA interactions. Gradient tree boosting algorithm is an effective machine learning-based method that has been successfully applied for both classification and regression problems [41–43].

In GTB algorithm, the decision function is initialized as:

8 $$ \Theta_{0} ({\chi}) = arg\ min_{c} \sum_{i=1}^{N} L(y_{i}, c),  $$

where *N* is the number of protein-lncRNA pairs in the training dataset. The gradient tree boosting algorithm repeatedly constructs *m* different classification trees *h*(*χ*,*α*_1_),*h*(*χ*,*α*_2_),...,*h*(*χ*,*α*_*m*_), each of which is trained based on a subset of randomly extracted samples, and then constructs the following additive function *Θ*_*m*_(*x*):

9 $$ \Theta_{m}({\chi}) = \Theta_{m-1}({\chi}) + \beta_{m}h({\chi}; {\alpha}_{m}),  $$

in which *β*_*m*_ and *α*_*m*_ are the weight and parameter vector of the *m*-th classification tree *h*(*χ*,*α*_*m*_). The loss function *L*(*y*,*Θ*_*m*_(*χ*)) is defined as:

10 $$ L(y, \Theta ({x})) = log(1 + exp(-y\Theta({\chi}))),  $$

where *y* is the real class label and *Θ*(*χ*) is the decision function. Both *β*_*m*_ and *α*_*m*_ are iteratively optimized by grid search so that the loss function *L*(*y*,*Θ*_*m*_(*χ*)) is minimized. Accordingly, we obtain the gradient tree boosting model $\tilde {\Theta }({\chi })$ as follows:

11 $$ \tilde{\Theta }({\chi}) = \Theta_{M}({\chi})  $$

We use grid search strategy to select the optimal parameters of GTB with 10-fold cross-validation on the benchmark dataset. The optimal number of trees of the GTB is 600, and the selected depth of the trees is 13. The rest parameters are set to default values.

## Results

### Training data sets

We randomly select 2,000 protein-lncRNA interactions from the experimentally validated protein-lncRNA associations as positive examples, and randomly generated 2,000, 4,000, 10,000, 20,000 negative samples that are not included in all known associations. As a result, we build a standard training set with 2,000 positive and 2,000 negative samples, and other three unbalanced data sets with more negative samples than positive ones. The ratios of positive and negative samples are 1:1, 1:2, 1:5 and 1:10 in the four training sets, respectively.

### Test data sets

For objective performance evaluation, an independent test set is built by randomly selecting 2,000 protein-lncRNA associations from the experimentally validated ones, plus 2,000 randomly generated negative samples. To be more realistic, we accordingly construct other three unbalanced test data sets with positive *vs* negative ratio 1:2, 1:5 and 1:10, respectively. Note that all the positive and negative samples in these test sets are independently chosen and excluded from the training set.

### Performance measures

We firstly evaluate the performance of our method using 10-fold cross-validation. The training set are randomly divided into ten set of roughly equal size subsets. Each subset is in turn used as the validation test data, and the remaining nine subsets are used as training data. The cross-validation process is repeated ten times, and the average performance measure over the ten folds are used for performance evaluation. We use multiple measures to evaluate the performance, including precision (PRE), recall (REC), F-score (FSC), accuracy (ACC) and the area under the receiver operating characteristic curve (AUC). They are defined as below:

$$precision=\frac{TP}{TP+FP},$$

$$Recall=\frac{TP}{TP+FN},$$

$$Accuracy=\frac{TP+TN}{TP+TN+FP+FN},$$

$$F-Measure=\frac{2\times Precision \times Recall}{Precision+Recall},$$

in which *TP* and *FP* represent the numbers of correctly predicted positive and negative samples, *FP* and *FN* represent the numbers of wrong predicted positive and negative samples, respectively. The AUC score is computed by varying the cutoff of the predicted scores from the smallest to the greatest value.

### Predictive power of topological features

**Fig. 2:**
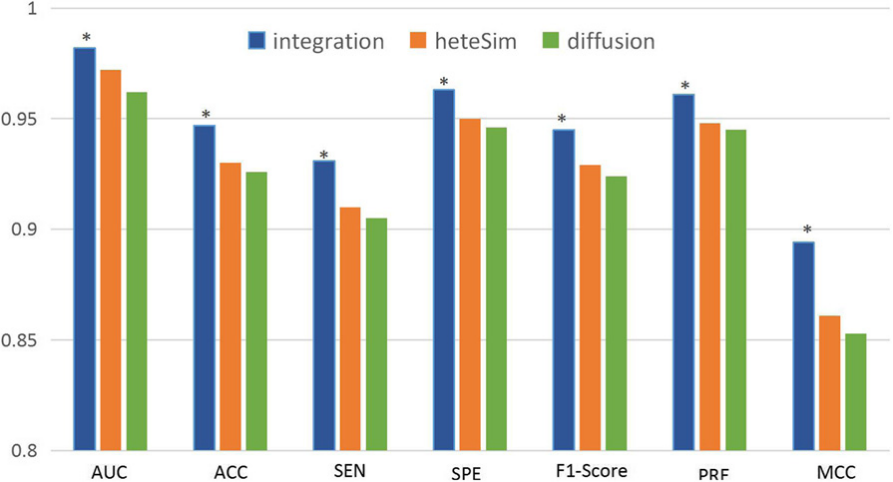
Performance comparison of different feature groups (Diffusion, HeteSim and combined feature)

To verify the effectiveness of the diffusion and HeteSim features in predicting protein-lncRNA interactions, we evaluate the predictive performance of the two feature groups alone and combination of them (combined features), on the standard training set. As shown in Fig. 2, the AUC values achieved by diffusion and HeteSim features are more than 0.97 and 0.96, respectively. The combined features obtains even higher performance, i.e. the AUC value reached 0.98. The experimental results show that the two types of topological features can accurately predict protein-lncRNA interactions. Moreover, the diffusion and HeteSim features are complementary and their combination can further improve the prediction performance.

### Benefit from gradient tree boosting algorithm

Since our method is based on the gradient tree boosting algorithm, we compared our method to several widely used classifiers, including k-nearest neighbors algorithm (kNN) [44], random forest (RF) [45] and support vector machine (SVM) [46], on our build standard training set using 10-fold cross validation. The counterpart classifiers are obtained from the python toolkits scikit-learn [47], and trained using the 1,014-dimensional combined features. For kNN classifier, we use 15 nearest neighbors and leaf size of 30 points. RF builds a number of decision tree classifiers trained on a set of randomly selected samples of the benchmark to improve the performance. A total number of 600 tree classifiers are built in this study. For SVM, we use radial basis function (RBF) as the kernel, and the penalty *c* and gamma *g* parameters are optimized to 512 and 0.00195, respectively. The number of trees used in the gradient tree boosting of PLIPCOM is set to 600, and the maximum tree depth is set to 13.

Table 2 show the prediction performance of PLIPCOM together with other methods. It can be found that PLIPCOM achieved the best performance with AUC, ACC, SEN, SPE, F1-Score and MCC of 0.982, 0.947, 0.931, 0.963, 0.946 and 0.895, respectively. The results indicate that the GTB algorithm substantially improves the overall performance.

**Table 2:** Performance comparison of GTB with other machine learning algorithms(k-NN, RF and SVM)

	AUC	ACC	SEN	SPE	F1-Score	MCC
KNN	0.916	0.860	0.871	0.849	0.862	0.721
RF	0.969	0.918	0.868	0.966	0.913	0.839
SVM	0.973	0.931	0.921	0.940	0.930	0.862
PLIPCOM	0.982	0.947	0.931	0.963	0.946	0.895

### Performance comparison with existing methods

**Fig. 3:**
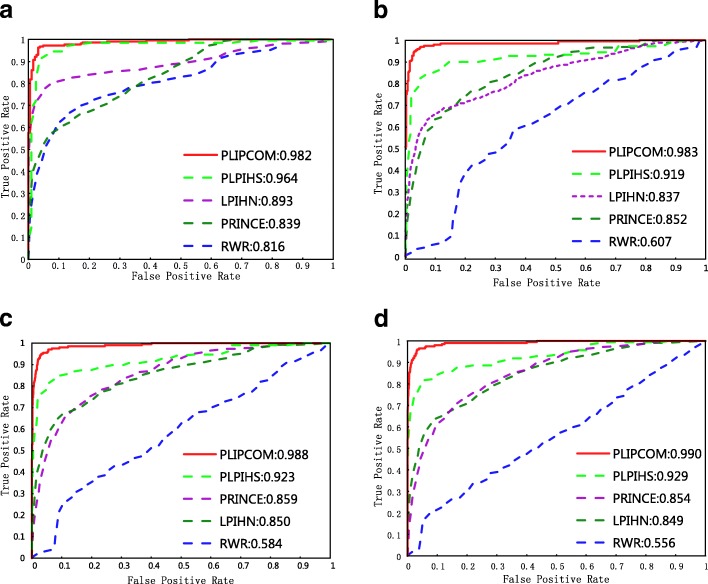
The ROC curves of PLIPCOM in comparison with other approaches on the train data sets with different positive and negative sample ratios. The four subfigures **abc** and **d** represent the ROC curves on the datasets with positive vs negative sample ratio 1:1, 1:2, 1:5 and 1:10, respectively

We compare PLIPCOM with four existing network-based prediction methods, including RWR [23], LPIHN [24], PRINCE [26] and PLPIHS [27], on the standard and three unbalanced data sets using 10-fold cross-validation. The parameter setting of PRINCE is that *α*=0.9, *c*=-15, *d*=log(9999) and the iteration number is set to 10. The parameters of LPIHN are set to their default values, i.e. *γ*=0.5, *β*=0.5 and *δ*=0.3. For RWR, the restart probability *r* is set to 0.5. The ROC curves are drawn using the true positive rate (TPR) *vs*. false positive rate (FPR) upon different thresholds of these prediction results. As shown in Fig. 3, PLIPCOM obtain the best performance among these protein-lncRNA interaction prediction methods, its AUC values achieved on four data sets are both more than 0.98. Particularly, the performance of PLIPCOM keeps stable on severely unbalanced data sets, while the performance of other methods is significantly influenced. For instance, on the ratio of 1:10 dataset, PLIPCOM achieved an AUC score of 0.990, and remarkably outperform PLPIHS (0.929), PRINCE (0.854), LPIHN (0.849) and RWR (0.556).

### Evaluation on independent test sets

**Fig. 4:**
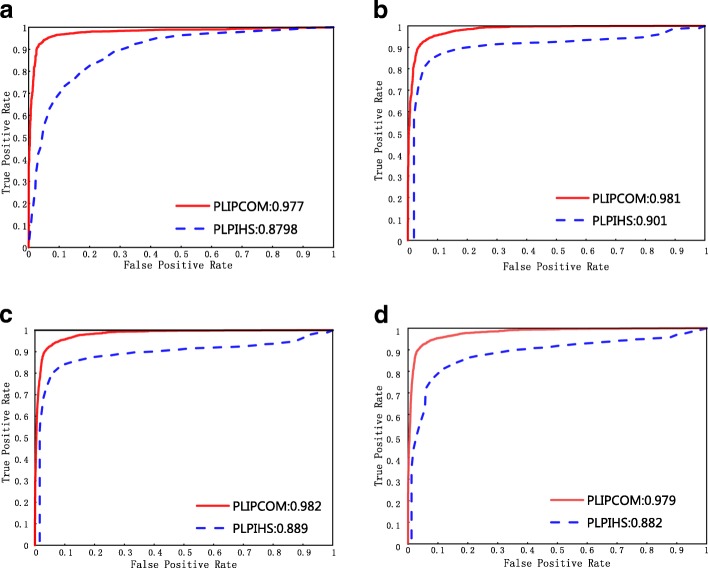
The ROC curves of PLIPCOM in comparison to PLPIHS on four test data sets with different positive and negative sample ratios. The four subfigures **abc** and **d** represent the ROC curves on the datasets with positive vs negative sample ratio 1:1, 1:2, 1:5 and 1:10, respectively

We further compare PLIPCOM with the most recent method, PLPIHS, on four independent test sets. As other three existing methods (PRINCE, LPIHN and RWR) are network-based and can only predict interactions between the nodes included in the prebuilt network, they can not work on independent test set and thus excluded out. In fact, PLPIHS has been shown to outperform other three existing methods in our previous study [27] and the aforementioned 10-fold cross validation. PLIPCOM and PLPIHS are trained on the standard training set, and then used to predict the protein-lncRNA interactions included in four independent test sets. We observed that PLIPCOM approach shows significant improvement compared with PLPIHS, as shown in Fig. 4. PLIPCOM achieved 0.977, 0.981, 0.982, 0.979 AUC score, which is much higher than 0.879, 0.901, 0.889, 0.882 by PLPIHS, on the independent test sets, respectively. It is worth noting that PLPIHS performs worse than PLIPCOM, mainly due to the fact that PLPIHS uses only the HeteSim features and a SVM classifier to predict protein-lncRNA interactions. The above results suggest that the two groups of topological features derived from the heterogeneous network are predictive of protein-lncRNA interactions, and their combination further improve the prediction performance.

### Case studies

To further illustrate the effectiveness of the proposed method, We present three lncRNAs for case studies, including HOTAIRM1 (ensemble ID: ENSG00000233429), XIST (ensemble ID:ENSG00000229807) and HOTAIR (ensemble ID:ENSG00000228630). The HOTAIRM1 is a long non-coding RNA that plays a critical role in regulating alternative splicing of endogenous target genes, and is also a myeloid lineage-specific ncRNA in myelopoiesis [48]. HOTAIRM1 locates between the human HOXA1 and HOXA2 genes. A multitude of evidence indicates that HOTAIRM1 play vital role in neural differentiation and is a potential diagnostic biomarkers of colorectal cancer [49]. The XIST encodes an RNA molecule that plays key roles in the choice of which X chromosome remains active, and in the initial spread and establishment of silencing on the inactive X chromosome [50]. HOTAIR is a long intervening non-coding RNA (lincRNA) whose expression is increased in pancreatic tumors compared to non-tumor tissue. Knockdown of HOTAIR (siHOTAIR) by RNA interference shows that HOTAIR plays an important role in pancreatic cancer cell invasion [51].

**Fig. 5:**
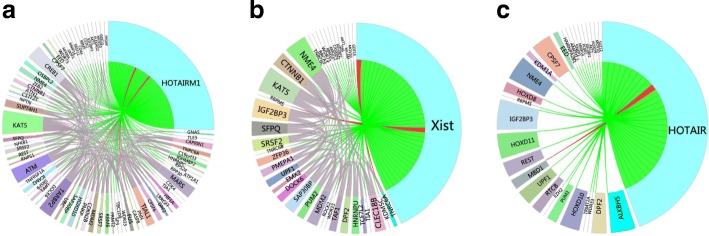
Prediction results of lncRNA HOTAIRM1, XIST, HOTAIR by PLIPCOM. (**a**), (**b**) and (**c**) show the results of HOTAIRM1, XIST, and HOTAIR, respectively. The correctly predicted interactions are colored in green between HOTAIRM1, XIST, HOTAIR and its partner genes, while wrongly predicted interactions are colored in red

In NPInter V3.0 [32], HOTAIRM1 is associated with 71 protein-coding genes, XIST is associated with 38 protein-coding genes and HOTAIR is associated with 29 protein-coding genes. We apply PLIPCOM to predict the interacting proteins of HOTAIRM1, XIST, HOTAIR and the results are shown in Fig. 5. Our method correctly predicted 69 interactions of HOTAIRM1, 36 interactions of HOTAIRM1, 28 interactions of HOTAIRM1. We further inspected top 10 predicted proteins of HOTAIRM1, XIST, HOTAIR as listed in Table 3. For example, GNAS protein is an imprinted region that gives rise to noncoding RNAs, HOTAIRM1, and other several transcripts, antisense transcripts that includes transcription of RNA encoding the *α*-subunit of the stimulatory G protein [52]. Indeed, GNAS has been shown to underlie some important quantitative traits in muscle mass and domestic mammals [53]. In addition, HOTAIRM1 can interact with SFPQ in colorectal cancer (CRC) tissues that release PTBP2 from the SFPQ or PTBP2 complex. The interaction between HOTAIRM1 and SFPQ is a promising diagnostic biomarker of colorectal cancer [54]. NFKB1 is a transcriptional factor that plays crucial role in the regulation of viral and cellular gene expressions [55], and its association with HOTAIRM1 is helpful to uncover the function of HOTAIRM1. Take HOTAIR for another example, EZH2 is the catalytic subunit of the polycomb repressive complex 2 (PRC2) and is involved in repressing gene expression through methylation of histone H3 on lysine 27 (H3K27) [56], EZH2 (predominant PRC2 complex component) inhibition blocked cell cycle progression in glioma cells, which is consistent with the effects elicited by HOTAIR siRNA. Through the study of EZH2, we can understand the biological function of HOTAIR more deeply [57]. These cases demonstrate that PLIPCOM is effective and reliable in predicting the interactions between lncRNAs and proteins.

**Table 3:** Top 10 ranked proteins for lncRNA HOTAIRM1, XIST and HOTAIR

lncRNA	Protein	Ensemble ID	Score
HOTAIRM1	GNAS	ENSG00000087460	0.978906
	NFKB1	ENSG00000109320	0.962423
	SFPQ	ENSG00000116560	0.956276
	PLEKHG2	ENSG00000090924	0.948234
	MMP14	ENSG00000157227	0.942456
	WDR73	ENSG00000177082	0.939295
	HNRNPC	ENSG00000092199	0.938295
	RPS24	ENSG00000138326	0.937062
	CPSF7	ENSG00000149532	0.936224
	SRSF11	ENSG00000116754	0.935515
XIST	GDF15	ENSG00000130513	0.98304
	NME4	ENSG00000103202	0.965669
	MOV10	ENSG00000155363	0.962258
	SFPQ	ENSG00000116560	0.961144
	QKI	ENSG00000112531	0.958775
	WDR73	ENSG00000177082	0.95635
	CASKIN2	ENSG00000177303	0.950001
	WDR33	ENSG00000136709	0.943944
	DPF2	ENSG00000133884	0.941258
	AKT1	ENSG00000142208	0.940658
HOTAIR	EZH2	ENSG00000106462	0.994214
	PUM2	ENSG00000055917	0.993374
	IGF2BP2	ENSG00000073792	0.970273
	UPF1	ENSG00000005007	0.965562
	PCBP1	ENSG00000169564	0.959887
	WDR33	ENSG00000136709	0.947819
	RTCB	ENSG00000100220	0.946163
	HNRNPA2B1	ENSG00000122566	0.945789
	SNIP1	ENSG00000163877	0.942754
	HOXD8	ENSG00000175879	0.93755

## Discussion and conclusion

Identification of the associations between long non-coding RNAs (lncRNAs) and protein-coding genes is essential for understanding the functional mechanism of lncRNAs. In this work, we introduced a machine learning method, PLIPCOM, to predict protein-lncRNA interactions. The major idea of PLIPCOM is to take full advantage of the topological feature of lncRNA-protein heterogenous network. We first build a protein-lncRNA heterogeneous network by integrating a variety of biological networks including lncRNA-lncRNA co-expression network, protein-protein interaction network, and protein-lncRNA association network. Two categories of features, including diffusion features and HeteSim features, are extracted from the global heterogeneous network. Subsequently, we apply the gradient tree boosting (GTB) algorithm to train the protein-lncRNA interaction prediction model using the diffusion and HeteSim features. Cross validations and independent tests are conducted to evaluate the performance of our method in comparison with other state-of-the-art approaches. Experimental results show that PLIPCOM gains superior performance compared to other state-of-the-art methods.

From our perspective, the superior performance of PLIPCOM benefits from at least three aspects: (i) diffusion features calculated using random walks with restart (RWR) on the protein-lncRNA heterogenous network, and the feature dimension is further reduced by applying singular value decomposition (SVD); (ii) HeteSim features obtained by computing the numbers of different paths from protein to lncRNA in the heterogenous network; and (iii) effective prediction model built by using the gradient tree boosting (GTB) algorithm. As far as our knowledge, we are the first to apply both diffusion and HeteSim features to predict protein-lncRNA interactions, although these two types features are regularly used in characterizing biological networks in previous works. As shown in our experimental results, diffusion and HeteSim features are complementary and their combination can further improve the predictive power. Moreover, compared to other classifiers, such as SVM and kNN, GTB used by PLIPCOM can not only achieve high prediction accuracy, but also select the feature of importance for identifying lncRNA-protein interactions.

The time complexity of our method depends mainly on the feature extraction procedure and GTB algorithm. The diffusion feature is calculated using RWR and its time complexity can be inferred from the equation *P*=(*E*−(1−*α*)*T*)^−1^(*αE*)=*αQ*^−1^*E*, in which *E* is unit matrix, *T* is the transition probability matrix, *α* is the restart probability and *Q* is an *n*∗*n* sparse matrix (*n* is number of nodes in the network). The time complexity of calculating inverse matrix *Q*^−1^ is *O*(*n*^3^), and can be optimized by using Cholesky algorithm. From our previous work, we know that the time complexity of calculating HeteSim feature is *O*(*kn*), where *k* is the number of samples and *n* is the number of nodes. Note that these two network features can be calculated in parallel. Moreover, we use the truncated SVD to reduce the diffusion feature dimension so that the time of GTB training process is greatly reduced. As a result, the time complexity of the methodology of PLIPCOM is moderate, and can be scaled to large networks.

Although PLIPCOM show effectiveness and promising predictive power, we think its performance can be further improved by adding protein sequence and structural information. In the near future, we will integrate sequence and structural features to promote the prediction of potential lncRNA-protein interactions.
